# Nucleotides-Induced Changes in the Mechanical Properties of Living Endothelial Cells and Astrocytes, Analyzed by Atomic Force Microscopy

**DOI:** 10.3390/ijms22020624

**Published:** 2021-01-10

**Authors:** Juan Carlos Gil-Redondo, Jagoba Iturri, Felipe Ortega, Raquel Pérez-Sen, Andreas Weber, María Teresa Miras-Portugal, José Luis Toca-Herrera, Esmerilda G. Delicado

**Affiliations:** 1 Departamento de Bioquímica y Biología Molecular, Facultad de Veterinaria, Instituto Universitario de Investigación en Neuroquímica (IUIN), Instituto de Investigación Sanitaria del Hospital Clínico San Carlos (IdiSSC), Universidad Complutense Madrid, 28040 Madrid, Spain; juan.gil-redondo@boku.ac.at (J.C.G.-R.); rpsen@ucm.es (R.P.-S.); mtmiras@ucm.es (M.T.M.-P.); 2 Department of Nanobiotechnology (DNBT), Institute for Biophysics, BOKU University for Natural Resources and Life Sciences, Muthgasse 11 (Simon Zeisel Haus), A-1190 Vienna, Austria; andreas.weber@boku.ac.at (A.W.); jose.toca-herrera@boku.ac.at (J.L.T.-H.)

**Keywords:** atomic force microscopy, astrocytes, endothelial cells, nucleotide receptor, P2Y nucleotide receptor

## Abstract

Endothelial cells and astrocytes preferentially express metabotropic P2Y nucleotide receptors, which are involved in the maintenance of vascular and neural function. Among these, P2Y_1_ and P2Y_2_ receptors appear as main actors, since their stimulation induces intracellular calcium mobilization and activates signaling cascades linked to cytoskeletal reorganization. In the present work, we have analyzed, by means of atomic force microscopy (AFM) in force spectroscopy mode, the mechanical response of human umbilical vein endothelial cells (HUVEC) and astrocytes upon 2MeSADP and UTP stimulation. This approach allows for simultaneous measurement of variations in factors such as Young’s modulus, maximum adhesion force and rupture event formation, which reflect the potential changes in both the stiffness and adhesiveness of the plasma membrane. The largest effect was observed in both endothelial cells and astrocytes after P2Y_2_ receptor stimulation with UTP. Such exposure to UTP doubled the Young’s modulus and reduced both the adhesion force and the number of rupture events. In astrocytes, 2MeSADP stimulation also had a remarkable effect on AFM parameters. Additional studies performed with the selective P2Y_1_ and P2Y_13_ receptor antagonists revealed that the 2MeSADP-induced mechanical changes were mediated by the P2Y_13_ receptor, although they were negatively modulated by P2Y_1_ receptor stimulation. Hence, our results demonstrate that AFM can be a very useful tool to evaluate functional native nucleotide receptors in living cells.

## 1. Introduction

Nucleotides are universal extracellular messengers that regulate nearly all bodily functions. Their actions are mediated by membrane receptors called P2 receptors, which are key pieces of the complex puzzle of purinergic signaling. Nucleotide receptors have been classified into two groups: ionotropic P2X receptors (P2XRs), which are ATP-gated cationic channels, and metabotropic P2Y receptors (P2YRs), belonging to the G-protein coupled receptor (GPCR) superfamily. These P2YRs consist of 7-membrane-spanning proteins coupled to G proteins and, in contrast to ionotropic receptors, P2YRs are selectively activated by adenine and uracil nucleotides, and even discriminate between tri- and diphosphate nucleotides [1,2].

So far, eight different P2YRs have been identified in mammals and are subdivided into two subgroups, based on phylogenetic and structural similarities: the “P2Y_1_ R-like” group, that includes P2Y_1_R, P2Y_2_R, P2Y_4_R, P2Y_6_R, and P2Y_11_R, which are coupled to Gq, and the “P2Y_12_R-like” subgroup, coupled to Gi, that includes the new ones P2Y_12_R, P2Y_13_R, and P2Y_14_R [3]. However, the most used classification of these receptors is based on their pharmacological profile, being ADP-selective receptors (P2Y_1_, P2Y_12_ and P2Y_13_ receptors), UTP/ATP-activated receptors (P2Y_2_R and murine P2Y_4_R) and UDP-selective receptor (P2Y_6_R). Within the P2Y family, the only specific receptor for ATP is the P2Y_11_R. The last identified P2Y_14_R is, in turn, activated by UDP-sugars. Overall, P2YRs are widely expressed in cells of all tissues and organs from neural and non-neural origin [2].

Stimulation of these metabotropic receptors triggers multiple intracellular signaling cascades that regulate cell differentiation, migration, proliferation and even cell death with physiopathological implications [2]. Any ongoing cellular process connects to changes in the mechanical properties of the cells. In this regard, Atomic Force Microscopy (AFM) has shown to be a useful tool for examining mechanical properties of adherent living cells, allowing the analysis of changes in the stiffness, deformability and adhesiveness of plasma membrane underlying cellular processes [4]. AFM delivers quantitative information about these parameters based on the indentation of the cell membrane under controlled location, rate and load. During the measurement, the indenting probe (of varying geometry, attached to the end of a cantilever) is moved towards the membrane by exclusively following the perpendicular Z axis, as the variation of the interacting forces between tip and the sample is measured, and later converted into force-versus-distance curves. From these, both the cell membrane stiffness and elasticity can be extracted. When a maximum load is reached, as defined at the beginning of the experiment, the motion of the tip is reversed and the force variation along the retraction path is monitored [5]. This reverse motion of the cantilever relates to the adhesive properties of the membrane, which can be quantified in terms of the maximum force required to detach the tip from the membrane. Additionally, the stepwise recovery of the basal (zero) force allows for the potential observation of rupture events and tethers, as a result of the gradual separation of the membrane from the tip, which provides information about the interaction between membrane and cytoskeleton. A joint consideration of these parameters offers an overview of the mechanical response featuring the cell behavior.

In the present study, we have analyzed whether nucleotide receptor stimulation induces noticeable changes of mechanical properties, both in human umbilical endothelial cells (HUVECs) and cerebellar astrocytes, by means of AFM in the so-called force spectroscopy mode. HUVECs have been used as a control cell line since they had been previously explored by AFM measurements [6,7]. Although endothelial cells and astrocytes are from different origin (mesoderm and ectoderm, respectively), they share some features like, for instance, their preferential expression of P2YRs, mainly P2Y_1_R, P2Y_2_R and P2Y_6_R [8,9,10]. In addition, both cell types are involved in tissue remodeling, in a process orchestrated by nucleotide receptors. Here, we have focused on the effects of P2YR stimulation, which can be selectively achieved by UTP challenges. P2Y_2_R is one of the receptors inducing cell migration in endothelial cells, macrophages, fibroblasts and astrocytes [11,12,13,14]. We have additionally tested the effect of 2MeSADP challenges for the stimulation of specific ADP receptors, P2Y_1_R, and P2Y_13_R subtypes. In endothelial cells, P2Y_1_R together with P2Y_2_R induce intracellular calcium mobilization and the release of NO and other bioactive molecules with important vasodilator actions [15,16]. In addition, it has been also proposed that endothelial and hepatocyte ADP receptors could also be involved in the reverse cholesterol transport [17]. In astrocytes and neurons, P2Y_1_R and P2Y_13_R play neuroprotective actions against different damage insults including hypoxic events, glutamate excitotoxicity and genotoxic stress, and also mediate pain transmission [18,19,20,21,22]. Hence, stimulation of nucleotide receptors in HUVEC cells, specially UTP-sensitive P2Y_2_ and P2Y_4_ receptors, increases cell membrane stiffness and membrane-cytoskeleton interaction, based on the data obtained from Young’s Modulus, maximum adhesive force and rupture event formation. A similar effect can be observed on astrocytes, since the stimulation of these cells with both UTP and 2MeSADP produces an increment on both cell membrane stiffness and adhesiveness, while also observing an enhancement in membrane-cytoskeleton connection. Furthermore, by using selective antagonists against P2Y_1_ and P2Y_13_ receptors we are capable to demonstrate that the effects caused by 2MeSADP on the mechanical properties of astrocytes directly depend on the activation of the P2Y_13_ receptor. Complementary analysis of the cell motility, by means of time-lapse videomicroscopy, confirmed the increase in cell motion due to 2MeSADP treatment, which became suppressed upon employment of MRS2211 for the selective blocking of P2Y_13_R. Then, AFM technique, and the mechanical approach, appears to be a sufficiently sensitive tool for elucidating both the presence of the nucleotide receptors, the effects induced by their respective activation, as well as to establish a proper distinction between them.

## 2. Results

### 2.1. Effects of P2Y Receptor Stimulation on HUVEC Mechanical Properties

**Figure 1:**
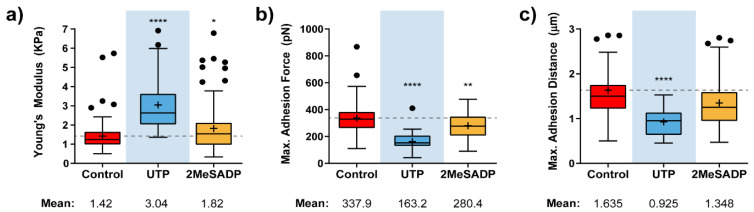
Effects of nucleotide stimulation of HUVECs on some mechanical parameters. Calculated instantaneous Elastic modulus (**a**) and adhesion-related (**b**,**c**) value variation for HUVEC cells (*N* > 15) exposed to UTP (blue), and 2MeSADP (yellow) agonists. Boxplots cover the range 25–75%, being the horizontal line indicative for the median, and the cross for the mean value, which are shown numerically below. For comparative purposes, a horizontal dashed grey line indicates control mean values. Whiskers indicate 5 and 95 %, while black dots refer to outliers. Statistically relevant variations *p* < 0.05, *p* < 0.01 and *p* < 0.0001 are highlighted by *, ** and ****, respectively.

HUVECs were treated, as described in our Methods section, with the maximal effective concentrations of UTP (100 µM) and 2MeSADP (10 µM) for the stimulation of P2Y_2_R and P2Y_1_R present in these endothelial cells, respectively [23,24]. AFM measurements on HUVECs stimulated with these P2Y agonists brought a significant variation for the particular case of cells exposed to UTP (Figure 1), whose elastic modulus doubles that from control cells (3.0 vs. 1.4 kPa, Figure 1a). This behavior relates to a considerable cell stiffening at the membrane level upon interaction with the nucleotide. Stimulation by UTP also reflected in the shape of a 2-fold decrease in the adhesion force (163 vs. 338 pN, Figure 1b), in comparison to untreated cells, and an evident shortening of the pulling distance required for observation of such a peak, which occurs roughly around 1µm (0.92 vs. 1.63 µm, Figure 1c). These effects speak about a less available membrane when the tip is retracted from the cell surface. The hypothesis supporting this behavior would be that the specific binding of UTP to P2Y_2_Rs might trigger the formation of a tighter connection between the membrane and the underlying cytoskeleton.

**Figure 2:**
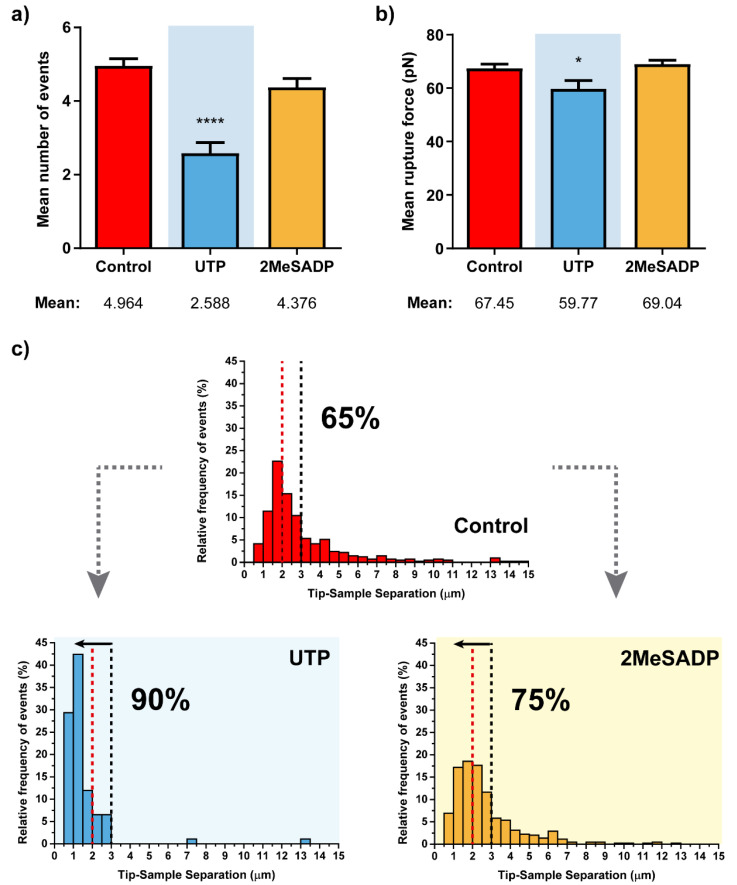
Rupture event and tether characterization for the respective treatments on HUVEC cells (*N* > 15). Figures (**a**,**b**) show the mean ±S.E.M. values for the number of tethers per curve and the rupture force. (**c**) Pulling distance- dependent relative frequency of appearance of full rupture events under the different conditions. Dashed lines highlight the maximum probability to find rupture events in control HUVEC cells (red) and the position at which observation of the 90% of the tethers in UTP-treated cells takes place, with the respective percentages in each case. Statistically relevant variations *p* < 0.05 and *p* < 0.0001 are highlighted by * and ****, respectively.

Such an assumption is reinforced when tending to the stepwise path followed by treated cells to achieve the zero-force (or full contact splitting with the indenting tip). There, the observation of a lower number of rupture events (Figure 2a), together with the fact that these tend to appear at much closer positions from the cell surface (up to the 90% of the ruptures taking place below a Z distance of 3 µm, Figure 2c), points in the same direction.

Although UTP appears to be the agonist inducing larger changes in the cells, or statistically more significant ones, HUVECs exposed to 2MeSADP also appeared to be influenced by this particular stimulation, indicating that ADP receptors present in these endothelial cells, preferentially P2Y_1_Rs, can also trigger a reaction from cells that follows a similar trend to that reflected upon UTP treatment. Thus, a slight increase of the mean Young’s modulus is detected (1.82 vs. 1.42 KPa), while both adhesion force (280.4 vs. 337.9 pN) and the appearing distance for the adhesion peak (1.348 vs. 1.635 µm) decrease.

Mainly, these first results provide a good indication of how sensitive and useful the use of the AFM in force mode can turn for unraveling the presence of nucleotide receptors and the nucleotide-induced mechanical responses in living cells. Once this possibility was confirmed on HUVECs, the next goal was to apply the same methodology on astrocytes exhibiting similar P2Y nucleotide receptor diversity.

### 2.2. UTP and ADP Sensitive Receptor Stimulation Modifies Mechanical Properties of Cerebellar Astrocytes

**Figure 3:**
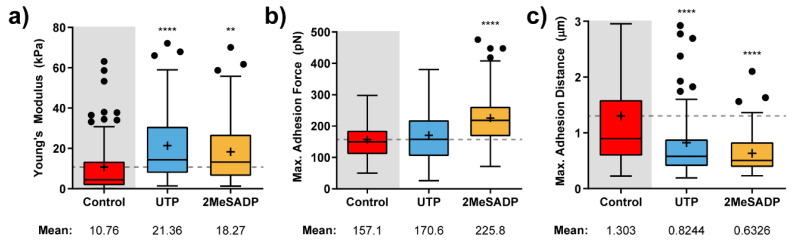
Effects of nucleotide stimulation of cerebellar astrocytes (*N* > 30) on the parameters determined with AFM. Calculated Elastic modulus (**a**) and adhesion-related (**b**,**c**) value variation for astrocytes exposed to 100 µM UTP (blue), and 10 µM 2MeSADP (yellow) agonists. Boxplots cover the range 25–75%, being the horizontal line indicative for the median, and the cross for the mean value, which are shown numerically below. For comparative purposes, a horizontal dashed grey line indicates control mean values. Whiskers indicate 5 and 95%, while black dots refer to outliers. Statistically relevant variations *p* < 0.01 and *p* < 0.0001 are highlighted by ** and ****, respectively.

Figure 3 shows the calculated elastic modulus and adhesion factor variations upon exposure of cerebellar astrocytes to UTP and 2MeSADP compounds. As expected, each of these agonists induces a mechanical response in glial cells that almost resembles what was previously observed in HUVECs, at least concerning the notorious stiffening taking place (around twice the control value, Figure 3a), and the appearance of the maximum force peak at much shorter pulling distances, with a drop around the 40 to 50% (Figure 3b,c).

The main difference would come from the particular impact each of the agonists has on the measured adhesion force. According to what is observed in Figure 3b, 2MeSADP has the highest impact (an increase of the 50%), although UTP slightly affected the mean adhesion force. In comparison to HUVECs, astrocytes might stick more in the beginning to the indenting tip, but as soon as the retraction motion goes on, the cytoskeleton effectively pulls in the opposite direction, and thus the adhesion peak is observed earlier.

**Figure 4:**
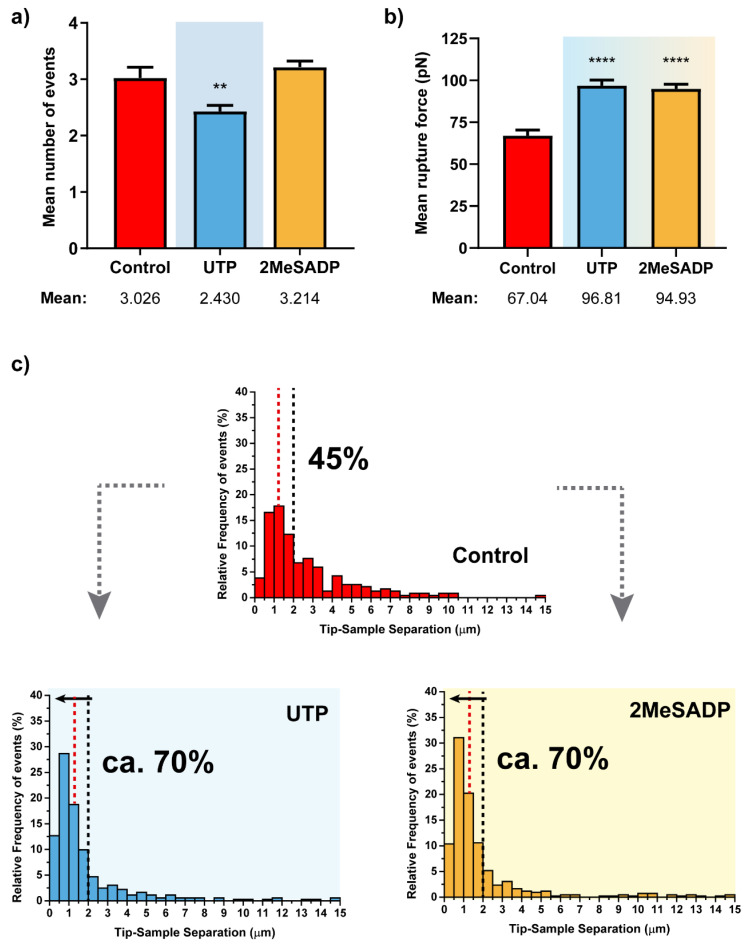
Rupture event and tether characterization for the respective UTP and 2MeSADP treatments on astrocytes (*N* > 30). Figures (**a**,**b**) show the mean ±S.E.M. values for the number of tethers per curve and the corresponding rupture forces. (**c**) Pulling distance- dependent relative frequency of appearance of full rupture events under the different conditions. Dashed lines highlight the maximum probability to find rupture events in control astrocytes (red) and the position at which observation of the 70% of the tethers in UTP-treated cells takes place, with the respective percentages in each case. Statistically relevant variations *p* < 0.01 and *p* < 0.0001 are highlighted by ** and ****, respectively.

From the remaining factors related to the retraction segment, a similar analysis can be extracted (Figure 4). Even though the number of rupture events does not show much variation, both mean rupture forces (Figure 4b) and distribution of the rupture event appearing distances (Figure 4c) indicate that the two agonists cause a clear response upon their receptor interaction. Hence, intensity of each rupture is significantly increased, and a higher majority of these events (70% vs. 45%) tends to be collected closer to the cell surface because of the previously mentioned potential reinforcement of the cell-cytoskeleton connection. Interestingly, individual responses to UTP and 2MeSADP are identical, so the response triggered by the corresponding nucleotide receptors is comparable.

These changes in cell mechanical properties and adhesion could be explained by cytoskeleton rearrangement [25]. One of the proteins implied in this rearrangement is the focal adhesion kinase (FAK). FAK is a member of non-receptor protein tyrosine kinases associated with integrins, focal adhesions and cytoskeletal proteins [26]. FAK was one of the first intracellular signaling proteins targeted by P2Y_2_ receptor in PC12 cells [27]. Later, it was demonstrated that P2Y_2_ receptor contains a RGD motif located in the first extracellular loop required for αV integrin interaction and migration induction in several cellular types, including HUVECs [11,28,29]. Considering that UTP also promotes migration in cerebellar astrocytes [14], we have analyzed by Western Blotting FAK contribution to cytoskeletal rearrangements underlying these mechanical changes. Stimulation of cerebellar astrocytes with either UTP or 2MeSADP induced a remarkable increase in the phosphorylated form (Tyr397) of FAK protein (Figure S1 Supporting information). The phosphorylation was detected at 5 min of stimulation and was maintained for up to 1 h with the nucleotide UTP, or 30 min in the case of 2MeSADP. The FAK phosphorylation pattern was similar to that previously observed in literature for HUVEC cells [11].

### 2.3. P2Y_13_ Receptor Mediates ADP Responses Detected by AFM in Cerebellar Astrocytes

**Figure 5:**
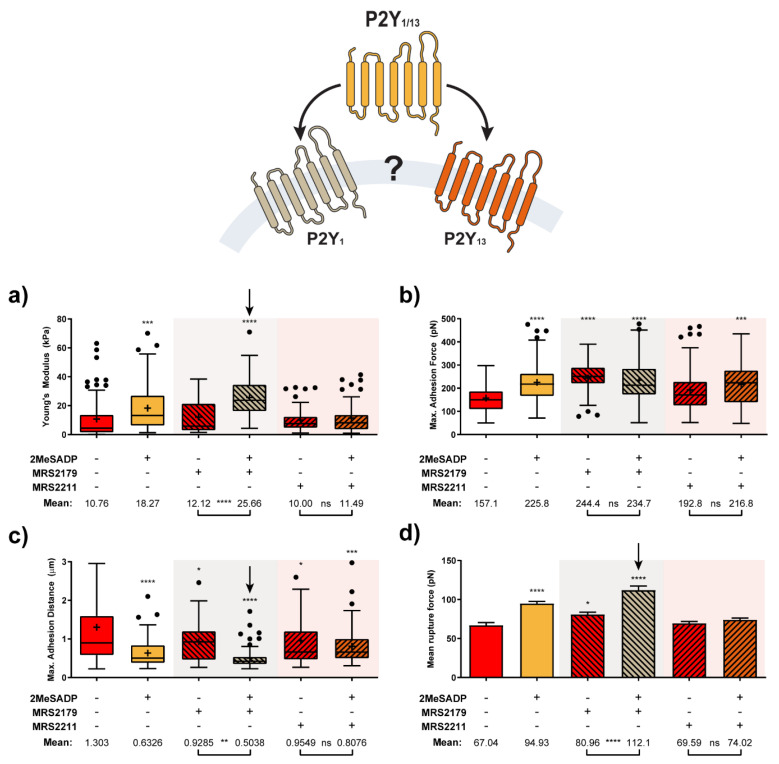
Characterization of ADP receptor mediating 2MeSADP effects observed in cerebellar astrocytes (*N* > 15). Calculated Elastic modulus (**a**) adhesion-related (**b**,**c**) and mean rupture force (**d**) value variation for astrocytes exposed to either MRS2179 and MRS2211 or their subsequent interaction with 2MeSADP receptor agonist. Boxplots (**a**–**c**) cover the range 25–75%, being the horizontal line indicative for the median, and the cross for the mean value, which are shown numerically below. Whiskers indicate 5 and 95%, while black dots refer to outliers. Columns in (**d**) indicate the mean ± S.E.M. of each condition. Statistically relevant variations *p* < 0.05, *p* < 0.01, *p* < 0.001 and *p* < 0.0001 are highlighted by *, **, *** and ****, respectively, while not significant variations (*p* > 0.05) are indicated by ns.

AFM measurements were taken to the subsequent level, so a more refined detection and identification of ADP receptors in charge of the reported 2MeSADP effects in astrocytes could be performed. With the help of specific antagonists, force measurements now aimed at discriminating between P2Y_1_ and P2Y_13_ receptors, which are distributed throughout the astrocyte population [30]. Both were considered so far as one single entity because of their similar affinity for 2MeSADP agonist. Thus, MRS2179 and MRS2211 antagonist compounds were employed to block P2Y_1_R and P2Y_13_R, respectively, and to examine their possible contribution based on the resulting mechanical response. Figure 5 depicts the variations collected for astrocytes in these conditions from both the approach and retraction segments.

**Figure 6:**
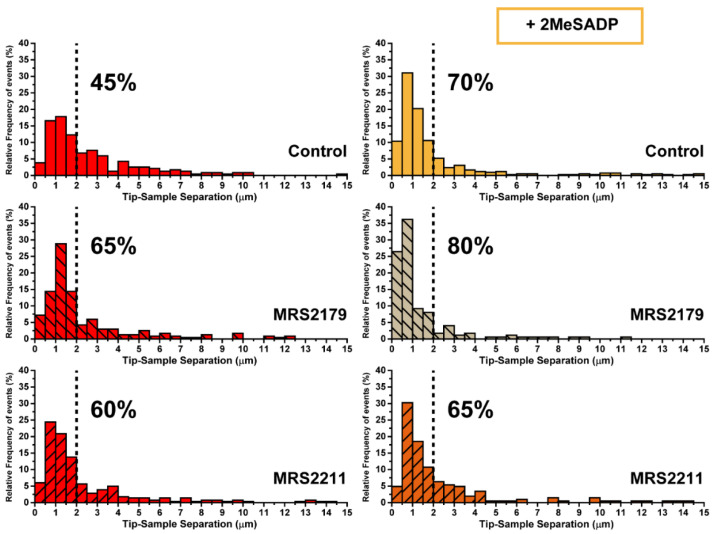
Characterization of ADP receptor mediating 2MeSADP effects observed in cerebellar astrocytes (II). Pulling distance-dependent relative frequency of appearance of full rupture for astrocytes (*N* > 15) exposed to either MRS2179 and MRS221 or their subsequent interaction with 2MeSADP receptor agonist. Dashed line highlights the position at which observation of the 70% of the tethers in 2MeSADP treated control cells takes place.

According to these results, treatment with the P2Y_13_R antagonist MRS2211 kept cells in a similar state to that shown by control astrocytes (non-stimulated cells). Non-significant effects were observed when 2MeSADP was added after MRS2211 treatment, since all the parameters under observation remained almost unaltered in comparison to values shown for MRS2211 on its own. In turn, for the case of pre-exposure to the specific P2Y_1_R antagonist, MRS2179, a mild cell reaction can be already identified, although the overall behavior did not differ much from control and MRS2211-treated astrocytes. Surprisingly, the effects caused by subsequent addition of 2MeSADP did not only match the trend recorded for unblocked (no antagonist treated) astrocytes, but the influence of such an addition became even more remarkable in some of the cases. This was indicated by the more extreme variations observed between unblocked and MRS2179-blocked cells in Figure 5: 18.27 vs. 25.66 kPa in elastic modulus, 225.8 vs. 234.7 pN in adhesion force, 0.633 vs. 0.504 µm in peak appearing distance, 94.9 vs. 112.1 pN in rupture forces, and a larger number of rupture events taking place at shorter distances (70% vs. 80% below 2 µm, see Figure 6). Apparently, pharmacological blocking of P2Y_1_R induces potentiation of P2Y_13_R responses, which reflects in the stronger mechanical response of the cell. On the contrary, since the blocking of P2Y_13_ receptors caused a total lack of response to 2MeSADP, the most plausible explanation points toward exclusive activity of this receptor in the 2MeSADP responses.

### 2.4. ADP Stimulation Alters Astrocyte Cell Motility through P2Y_13_ Receptor Activation

**Figure 7:**
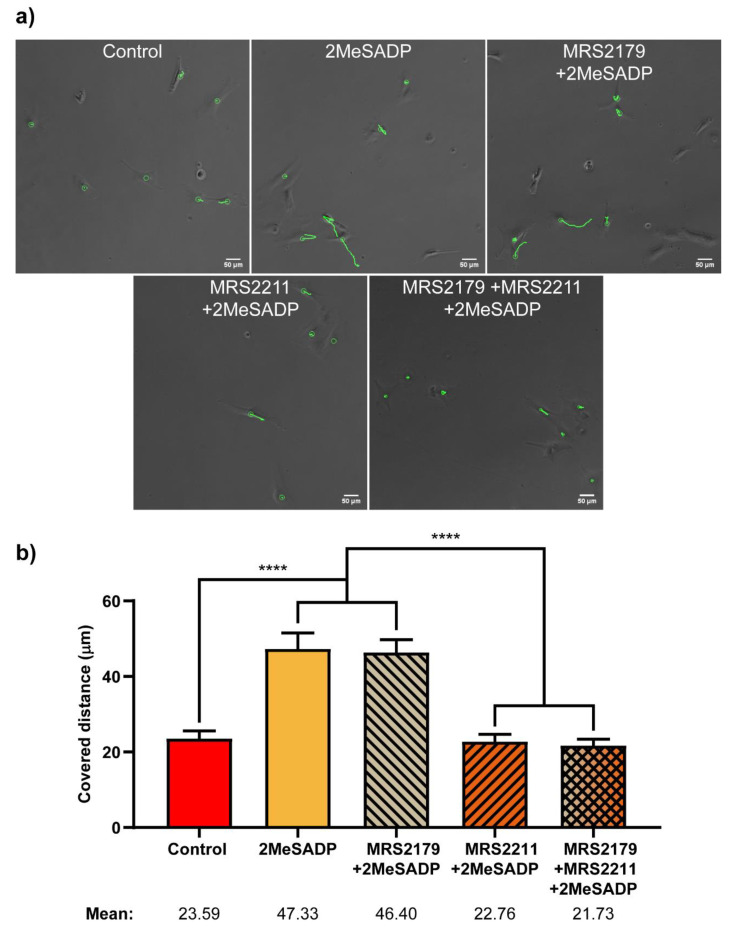
Effects of 2MeSADP treatment and P2Y_1_ and P2Y_13_ antagonism on migratory capabilities of rat cerebellar astrocytes. (**a**) Bright field images of astrocytes obtained after three hours of Time-Lapse video microscopy. Green circles indicate different tracked cells. Green lines show the location of the cell during the three hours. (**b**) Columns show the total distance covered by the cells, in microns, during a period of three hours, in basal conditions or after incubation with 2MeSADP for one hour, in the presence or absence of antagonists MRS2211 and MRS2179, and a combination of both. Whiskers indicate S.E.M., statistically significant differences are indicated by **** when *p* < 0.0001.

Complementarily to AFM experiments, the motility of cerebellar astrocytes was tested upon ADP receptor stimulation. As mentioned above, P2Y_13_R stimulation in cerebellar granule neurons and astrocytes displays neuroprotection [31,32]. P2Y_1_R also exerts neuroprotective actions in astrocytes from other brain areas [18,19,33]. However, ADP receptors, P2Y_1_, P2Y_12_ and P2Y_13_ also mediate migration and proliferation [34,35]. In order to analyze the effects of ADP receptors on cell migration, astrocytes were stimulated with 10 µM 2MeSADP for one hour either in the presence or absence of selective antagonists of P2Y_13_ and P2Y_1_ receptors (compounds MRS2211 and MRS2179, respectively), and the distance covered by cells during the following three hours was measured by means of time-lapse video-microscopy. As shown in Figure 7, treatment of astrocytes with 2MeSADP increased the distance covered by the cells during the three hours rather significantly, an effect that was completely abolished in the presence of P2Y_13_ selective antagonist MRS2211. P2Y_1_ selective antagonist MRS2179, however, proved unable to suppress the effects of 2MeSADP on cell movement. The same behavior was observed when exposing cells to both antagonists, which discarded analysis of this system by AFM. Thus, these results confirm that 2MeSADP effects on astrocytes are a straightforward consequence of P2Y_13_ receptor stimulation, rather than of P2Y_1_.

## 3. Discussion

The main goal of this work was to investigate the responses evoked after nucleotide receptor stimulation with a new methodological approach, Atomic Force Microscopy, which allows for the analysis of changes in the mechanical properties of the membrane in living cells at the nanometric level.

We have shown that stimulation of nucleotide receptors in the two cell types examined, HUVEC and cerebellar astrocytes, promotes changes in the mechanical parameters, as determined by AFM. Those changes are reflected as variations in the stiffness, viscosity and elasticity of the plasma membrane. In HUVECs the most relevant changes were observed after UTP-sensitive receptor stimulation. However, UTP- and ADP-sensitive receptor stimulation in astrocytes affected AFM parameters rather equally. These effects are not surprising, as nucleotide receptors interact and activate integrins [28,29,36]. Integrins are dimeric transmembrane proteins consisting of α and β subunits, that act as signaling molecules between the plasma membrane and extracellular matrix. Moreover, integrins are inner attached to actin filaments and mechanically stabilize the plasma membrane contributing to the maintenance of the cellular morphology and function. Thus, nucleotide receptor activation could modify these interactions inducing cytoskeleton rearrangements. The main target of UTP is P2Y_2_R, which mediate migration process in different cellular types including endothelial cells and astrocytes [11,14]. Migration is a dynamic process that requires a chained contribution of diverse cell constituents, and that has an obvious impact on the mechanical response of the cell.

As aforementioned, employment of a technique like Atomic Force Microscopy enables quantification of the ongoing changes in mechanics-related factors (i.e., elastic modulus, adhesion force, etc.) with high sensitivity and values falling in the nanoscale [37]. Approaches so far regarding astrocytes are scarce, but they have already pointed toward the potential application of this technique. First experimental examples dealt with the contribution of the cytoskeleton to the elastic response of the cell, mainly attending to its integrity [38], arrangement [39], and maturation levels [40]. The latter, for example, indicated that the positive change of stiffness is age-dependent. Attending to those results, nanomechanical changes observed in astrocytes correlate with the maturation of intracellular structures, in particular that of cytoskeletal elements, which turns out to be very helpful in predicting the in-vivo function of these glial cells. In their results, authors have found out how the Young’s modulus could shift from 3.8 to above 20 kPa only in a week time, and far above for older specimens, in agreement with the morphological changes observed. This conclusion represents a good reference for comparative purposes and underlines the usefulness of AFM results as an analytical and diagnostic tool.

In our experiments, the mechanical behaviour of astrocytes (from comparable early age) was measured upon selective activation of P2Y_2_ and P2Y_1/13_ receptors by the respective agonists (UTP and 2MeSADP). Values were compared to those from endothelial HUVEC cells, being a cell line extensively characterized in previous mechanical studies [41,42], and known their relatively similar receptor expression. In general, results show a synchronized variation of the mechanical factors under control: Young’s modulus, Adhesion Force and Maximum adhesion distance, and in the formation of rupture events as the indenting tip retracts. These changes indicate a straightforward connection between receptor stimulation and the mechanical response triggered. As could be observed, P2YR stimulation induces remarkable cell stiffening and a tighter membrane-cytoskeleton connection, independently of the cell type under analysis. The latter is featured by the significant drop in the distance required for the appearance of the maximum adhesion force, together with the larger number of rupture events at short distances upon membrane pulling. Interestingly, P2Y_2_ receptor activation achieved with UTP in both HUVEC and astrocytes led to comparable responses: 2-fold increase in the Young’s modulus, appearance of the adhesion peak around a 40% closer and same percentage of rupture events displaced towards short retraction distances (25%). Simultaneously, HUVEC and astrocytes differ in their relative adhesion forces measured (maximum peak and rupture), on which the specific particularities featuring each membrane (local charge, etc.) might also play a critical role on their respective interaction with the indenting probe. Mechanical strain, as induced in these experiments, has also been identified among the factors that might affect the reaction level in astrocytes, which can derive into different expression of transmembrane proteins or the release of various molecules [43]. In any case, and leaving this fact aside, P2Y_2_ receptor stimulation by UTP shows to truly influence the unbinding adhesion (either increased or decreased) in comparison to that between the tip and their corresponding controls. For the case of ADP receptor activation by 2MeSADP challenges, such an overall mechanical alteration does also exist, though to a very different extent. Indeed, for HUVEC cells it is very subtle, almost non-existing, and might indicate a lower expression level of these receptors. However, stimulation of ADP receptors with the previous available agonist, 2MeSATP, also induced calcium responses, which affected membrane permeability. In this case, P2Y_1_R appears to be involved [44]. Contrarily, 2MeSADP challenges have a rather strong impact on astrocytes in a similar manner to that induced by UTP for both the elastic modulus (2-fold increase), and the appearing distances of both the adhesion peak (60%) and that of the rupture events. Actually, distribution of these events is almost identical, with around the 70% taking place below 2 µm of pulling (45% for control astrocytes). In previous studies, we demonstrated that P2Y_2_ and P2Y_1_/P2Y_13_ receptors share some intracellular signalling cascades in cerebellar astrocytes. Their stimulation induces intracellular calcium mobilization and ERK1/2 activation, although it differs in the activation pattern. ERK activation displayed by UTP receptors was more sustained than those triggered by ADP receptors [45]. In this line, results reported here indicate that these receptors also share another key protein linked to cytoskeleton rearrangement, Focal Adhesion Kinase (FAK). UTP and 2MeSADP challenges increase FAK phosphorylation at Tyr397. This residue is autophosphorylated after FAK activation and acts as a binding site for other intracellular signalling proteins, like src-like kinases and PI-3 kinases, well-known targets of nucleotide receptors in astrocytes and endothelial cells [11,14,46].

An open question in the mechanical response recorded for exposure to 2MeSADP would be whether it exclusively comes from activation of P2Y_1_R, solely from P2Y_13_R, or if it derives from a joint action of both receptors. Attending to the sensitivity shown by atomic force microscopy up to this point, such a hypothesis was tackled by selectively blocking each of the receptors involved with the corresponding antagonists (MRS2179 and MRS2211, respectively), and by comparing the mechanical response obtained for those astrocytes upon new exposure to 2MeSADP. Blocking of P2Y_13_ receptors showed the effective cancellation of the mechanical response of astrocytes upon subsequent exposure to 2MeSADP, indicative of its exclusive activity. On the contrary, blocking of P2Y_1_R potentiated the influence of 2MeSADP on the cell mechanics. This observation points to the fact that in response to P2Y_1_R suppression, the activity of P2Y_13_R is reinforced and cells can still behave as those without antagonist treatment (see Figure 7). A similar cross talk was described for two ADP receptors, P2Y_1_ and P2Y_12_, present in platelets [47]. These authors demonstrated that P2Y_1_R inhibits P2Y_12_ signalling through Src kinase, and a reciprocal feedback pathway operates whereby P2Y_12_R positively regulate calcium responses elicited by P2Y_1_R.

As mentioned above, UTP stimulation induced cell migration in astrocytes [14,46]. Migration studies had been accomplished by transwell migration and wound-healing assays. In turn, 2MeSADP stimulation had no effect. However, considering that ADP receptors have been also involved in microglial chemotaxis [34] and astroglial proliferation [35], we have analysed cell behaviour after 2MeSADP stimulation in astrocytes plated at low density, in the same experimental conditions used in AFM experiments. Tracking of astrocytes by time-lapse video microscopy revealed that P2Y_13_R stimulation increased cell motility at least during the three hours analysed. All these findings confirm that stimulation of nucleotide receptors can trigger multiple actions depending on cellular context, microenvironment, cell density, duration of the stimulation, and in certain extent also depend on the type of assays used.

To our knowledge, this could be the first time that mechanics-based AFM approaches have been employed to resolve the receptor contribution in primary cultures (rat cerebellar astrocyte cultures). In literature, previous receptor recognition approaches involving the use of AFM focused on its use as molecular recognition imaging device, after specifically decorating each receptor subunit with histidine (His) tags in a cell line [48,49]. Alternative studies employed fast-scanning AFM devices to even follow conformational changes in single receptors [50]. Then, the success of the obtained results opens the door to application of this methodology for discriminative native receptor recognition experiments, and to further evaluation of the mechanical response of selectively stimulated astrocytes, something authors are currently developing. This approach also uncovers the implication of the different intracellular signals activated by nucleotides in the changes of the mechanical properties of the plasma membrane.

Undoubtedly, these studies could be applied to other nucleotide receptors. Ionotropic P2X and metabotropic P2Y nucleotide receptors are involved in mechanotransduction in different tissues in physiological conditions or after injury or inflammatory processes [32,51,52,53,54,55,56].

## 4. Materials and Methods 

### 4.1. Chemicals, Materials and Antibodies

Papain was purchased from Worthington (Lake Wood, NJ, USA), fetal bovine serum (FBS) and other culture reagents were obtained from GIBCO (Life Technologies, Barcelona, Spain). Plastic Petri dishes and culture flasks were supplied by Falcon Becton Dickinson Labware (Franklin Lakes, NJ, USA). Borosilicate circular cover glasses (diameter: 24 mm, 15 mm, thickness: 0.08–0.12 mm) were from Menzel Gläser, VWR, (Bruchsal, Germany). Oxygen plasma was from Gala Instruments GmbH, (Bad Schwalbach, Germany). Antibiotics, bovine serum albumin (BSA), Dulbecco’s Modified Eagle Medium (DMEM), L-15 Medium (Leibovitz), MRS 2179 (2′-Deoxy-N6-methyl adenosine 3′,5′-diphosphate), MRS 2211(2-[(2-Chloro-5-nitrophenyl)azo]-5-hydroxy-6-methyl-3-[(phosphonooxy)methyl]-4-pyridinecarboxaldehyde), nucleotides, and antisera for GAPDH were purchased from Sigma Aldrich (St. Louis, MO, USA). Specific antibodies against phospho-FAK (Tyr397) and FAK were purchased from Elabscience Biotechnology Inc. (Houston, TX, USA). Secondary horseradish peroxidase-conjugated anti-rabbit antibodies were from Dako (Glostrup, Denmark). All other non-specified reagents were routinely supplied by Sigma, Merck (Darmstadt, Germany) or Roche Diagnostics SL (Barcelona, Spain).

### 4.2. Cell Cultures 

HUVEC cells employed in this work were kindly provided by Dr. Spela Zemljic-Jokhadar (Medical Faculty, University of Ljubjana, Ljubljana, Slovenia). Cells were grown until confluence in culture flasks in DMEM medium supplemented with 10% FBS, 2 mM glutamine, 100 U/mL penicillin, and 100 μg/mL streptomycin. Cell cultures were maintained at 37 °C in humidified atmosphere containing 5% CO_2_.

Primary cultures of cerebellar astrocytes were prepared as described previously [57]. These experiments involving animals were carried out at the Complutense University of Madrid (Madrid, Spain), following the International Council for Laboratory Animal Science guidelines. Briefly, the cerebella were removed aseptically from Wistar rat pups (P7) and digested with papain. The so-obtained cerebellar dissociated cells were resuspended in DMEM containing 10% (*v*/*v*) FBS, 25 mM glucose, 2 mM glutamine, 100 U/mL penicillin, 100 mg/mL streptomycin and 2.5 µg/mL amphotericin, and they were plated in culture flasks at a density of 70,000 cells/cm^2^. The cells were maintained in culture until they reached confluence (approximately 10–12 days), replacing the medium every 3–4 days. Purified astrocyte cultures were obtained by orbital shaking. Astrocytes were then detached from the culture flasks by trypsin digestion and cryopreserved in FBS containing 10% DMSO at −80 °C.

The thawing of cellular vials was made in a 37 °C water bath, and the cells were immediately resuspended in DMEM culture medium and collected by centrifugation (at 150× *g* for 10 min) at room temperature. Finally, astrocytes were cultured in culture flasks as mentioned above to reach confluence.

### 4.3. Atomic Force Microscopy

For AFM experiments, confluent cultured cells, HUVECs or astrocytes, were detached from culture flasks and plated onto 24 mm diameter glass coverslips in 6-well multiwell plates in culture medium supplemented with glutamine, antibiotics and 10% FBS at a density of 4000 cells/cm^2^, and they were used two days after plating. Prior to incubation, coverslips were rinsed with EtOH, N_2_ dried, and cleaned with oxygen plasma (GaLa Instrumente GmbH, Bad Schwalbach, Germany). Cells were first washed with fresh Leibovitz medium for 30 min for the trophic factor withdrawal and stimulated with the indicated nucleotides in the same medium for 1 h at 37 °C by using a custom thermo-regulated flow-cell (JPK Instruments, Berlin, Germany). AFM measurements were accomplished with a Nanowizard III instrument (JPK Instruments, Berlin, Germany) incorporating a Cell Hesion module (which allows the extension of the piezo Z range to 100 µm), mounted on an inverted optical microscope (Axio Observer Z1, Zeiss, Jena, Germany). The AFM was operated in force-spectroscopy mode, applying constant setpoint (1.5 nN) and loading rate (5 µm/s), as previously described [58]. These values were chosen to avoid an influence on the cell mechanics outcome, as recently described by Weber et al. [41]. Cantilevers equipped with a quadratic pyramidal tip (r = 22 nm, half-to-face-angle = 35) were calibrated before each experiment by means of a thermal tune method (calculated k values ranging between 0.14 and 0.19 N/m) and compared to the nominal spring constant of the cantilever (k = 0.12 N/m). Cells were always indented above the nucleus to reduce variability and substrate artifacts. Each cell was indented ten times to reach a sufficient amount of plots for statistical purposes. Between the indentation of cells, the substrate was probed to ensure tip cleanliness.

### 4.4. Data Analysis

Force curves were analyzed using JPKSPM Data Processing software (JPK Instruments, Berlin, Germany). Briefly, deflection and distance of the cantilever were transformed into force-versus-distance curves after specifying previously measured values of spring constant of the cantilever and photodiode sensitivity. Basal line and contact point were then determined on approach curves, and Young’s Modulus values were obtained after fitting of the curve following a Hertz-Sneddon model optimized for the tip geometry employed (quadratic pyramid), according to Equation (1):

(1) $$F=\frac{E}{1-\upsilon }\frac{\tan\left(\alpha \right)}{\sqrt{2}}\delta^{2}$$

where *E* is the Young’s Modulus, *ν* is the Poisson’s ratio (set to 0.5 assuming cells are incompressible), *α* is the face angle of the pyramid (22°), and *δ* is the indentation. An indentation of 350 nm (corresponding to less than 10% of the cell height) was used to calculate the Young’s Modulus.

Retract curves were adapted similarly and maximum adhesion force was measured as the lowest point of the curve in the Y axis. The total number of rupture events, their relative position (being considered as zero the Z position at which tip retraction starts after system relaxes for 10 s) and the rupture force associated were also saved for further analysis. Examples of representative approach and retraction plots can be seen in the supporting information (Figure S2).

### 4.5. Western Blotting

Total cellular lysates from astrocytes plated onto Petri dishes were obtained as previously described [57], and aliquots (15–20 μg protein) were resolved by SDS-PAGE and transferred to PVDF membranes. Membranes were blocked in BSA dissolved in PBS (3% *w*/*v*), and then incubated overnight at 4 °C with primary antibodies diluted in bovine serum albumin (3% BSA *w*/*v*) dissolved in PBS containing 0.1% Tween-20. The primary antibodies were used at the following dilutions: anti-phospho-FAK (Tyr397), anti-total FAK (1:1000) and anti-GAPDH (1:10,000). Antibody binding was detected for 1 h at room temperature with anti-rabbit (1:1000) horseradish peroxidase-conjugated secondary antibodies and visualized by the ECL method (kit Western Lighting ECL PRO, Perkin Elmer, Madrid, Spain). Chemiluminescence images were obtained with the ImageQuant LAS 500^®^ image system and quantified by densitometry using the ImageQuantTL software.

### 4.6. Time-Lapse Video Microscopy and Single Cell Tracking

For time-lapse video microscopy, astrocytes were plated onto 15 mm coverslips at the same density used for AFM experiments and used the following day after plating. They were stimulated with 10 µM 2MeSADP for one hour, followed by time-lapse video microscopy and single cell tracking with a NIKON TE-2000 microscope at a constant temperature of 37 °C and in 5% CO_2_ [59]. Images were acquired every 5 min over 3 h using a long-distance 20× phase contrast objective (Nikon), a ZYLA camera from ANDOR and the 4.7/NIS-elements software from NIKON. Cell speed was analyzed by single-cell tracking using software prepared ad-hoc (The Tracking Tool, TTT) [60]. Finally, cells were immunostained with anti GFAP antibody. Where indicated, cells were pretreated with P2Y antagonists for 5 min before the nucleotide addition. Migratory capabilities of astrocytes were measured as the total distance covered during three hours of time-lapse.

### 4.7. Statistical Analysis

Statistical analysis of AFM data was carried out using Graphpad Prism 6 (GraphPad Software Inc., San Diego, CA, USA) and Origin Pro 9 (OriginLab, Northhampton, MA, USA) programs. Young’s Modulus values, maximum adhesive force, mean number of rupture events and mean rupture force are expressed as the means ± S.E.M. calculated from the force curves obtained from three experiments performed from different cultures. Each experiment consisted on ten measurements per cell, over ten cells from each experimental condition.

Migratory capabilities of astrocytes are expressed as the total distance covered by ten different cells, from ten different fields for each experimental condition. Results are expressed as the means ± S.E.M.

Statistical differences between two groups of data were assessed using a *t* test and a *p* value < 0.05 was taken as the limit of significance. When multiple comparisons were made, one-way analysis of variance was used and a Dunnett’s post-test analysis was applied only when a significant (*p* < 0.05) effect was evident.

## 5. Conclusions

In this work, we have employed atomic force microscopy to characterize, for the first time, the mechanical response of astrocytes on the nanoscale, upon selective stimulation of purinergic metabotropic receptors P2Y_2_ and P2Y_1/13_ with the respective agonist (UTP and 2MeSADP). Results have shown a clear synchronized variation in several mechanical factors (Young’s modulus, adhesion, rupture event formation) whose intensity varied depending on the receptor expression levels found in cells. Overall, stimulation induces astrocyte stiffening and a tighter connection between the cytoskeleton and the membrane, with special relevance for the use of UTP (P2Y_2_R). In a second step, the corresponding contribution of P2Y_1_ and P2Y_13_ receptors to the mechanical response obtained through the use of 2MeSADP agonist has been unraveled. This became possible by selectively blocking each receptor with the respective antagonists MRS2179 and MRS2211 before astrocyte exposure to the agonist molecule. Evaluation of the mechanical data has shown suppression of the cellular response only upon the blocking of P2Y_13_R, which indicates that this receptor is the one in charge of the stimulation-induced changes in the mechanical properties. Hence, the overall sensitivity shown by the mechanical approach under atomic force microscopy technique reveals it as a suitable tool for the detection and characterization of additional purinergic receptors in astrocytes (and additional cell lines), which will become a main target of our future research works.

## Data Availability

Not applicable.

## Supplementary Materials

The following are available online at https://www.mdpi.com/1422-0067/22/2/624/s1. Figure S1: FAK phosphorylation induced by the UTP and 2MeSADP stimulation in rat cerebellar astrocytes. Representative blots showing changes in phopsho-FAK (Tyr 397) protein levels after treatment with either 100 µM UTP or 10 µM 2MeSADP for different periods of time are shown, together with total FAK and GAPDH blots, Figure S2: Representative examples of approach (a) and retract (b) plots obtained from Force measurements. Figure a shows the comparison between the average curves for astrocytes without (control, red) and with exposure to agonist molecules UTP (Blue) and 2MeSADP (yellow). The inset shows a magnified view of the area highlighted by the dashed grey box, which corresponds to the section of the curve employed for the calculation of the respective Young’s moduli (initial 300 nm of the indentation). (b) Retraction plot showing the pulling distance-dependent adhesion-related factors (Adhesion Force, event rupture force and appearing distance). The rupture steps are highlighted in red.
