# Supplementary material for: Nucleotides-Induced Changes in the Mechanical Properties of Living Endothelial Cells and Astrocytes, Analyzed by Atomic Force Microscopy
Source: Int J Mol Sci. 2021 Jan 10;22(2):624. doi: 10.3390/ijms22020624 (PMC7827192; doi:10.3390/ijms22020624)
Supplement: Supplementary file 1 [file ijms-22-00624-s001.pdf]

International Journal of
Molecular Sciences

MDPI

Supporting Information

Nucleotides-Induced Changes in the Mechanical Properties of Living Endothelial Cells and Astrocytes, Analyzed by Atomic Force Microscopy

Juan Carlos Gil-Redondo [1,2], Jagoba Iturri [2,*], Felipe Ortega [1,*], Raquel Pérez-Sen [1], Andreas Weber [2], María Teresa Miras-Portugal [1], José Luis Toca-Herrera [2] and Esmerilda G. Delicado [1,*]

[1] Departamento de Bioquímica y Biología Molecular, Facultad de Veterinaria, Instituto Universitario de Investigación en Neuroquímica (IUIN), Instituto de Investigación Sanitaria del Hospital Clínico San Carlos (IdiSSC), Universidad Complutense Madrid, 28040 Madrid, Spain; juan.gil-redondo@boku.ac.at (J.C.G.-R.); rpsen@ucm.es (R.P.-S.); mtmiras@ucm.es (M.T.M.-P.)

[2] Institute for Biophysics, Department of Nanobiotechnology (DNBT), BOKU University for Natural Resources and Life Sciences, Muthgasse 11 (Simon Zeisel Haus), A-1190 Vienna, Austria; andreas.weber@boku.ac.at (A.W.); jose.toca-herrera@boku.ac.at (J.L.T.-H.)

* Correspondence: jagoba.iturri@boku.ac.at (J.I.); fortegao@ucm.es (F.O.); esmerild@ucm.es (E.G.D.); Tel.: +43-1-47654-80354 (J.I.); +34-91-394-3892 (E.G.D.)

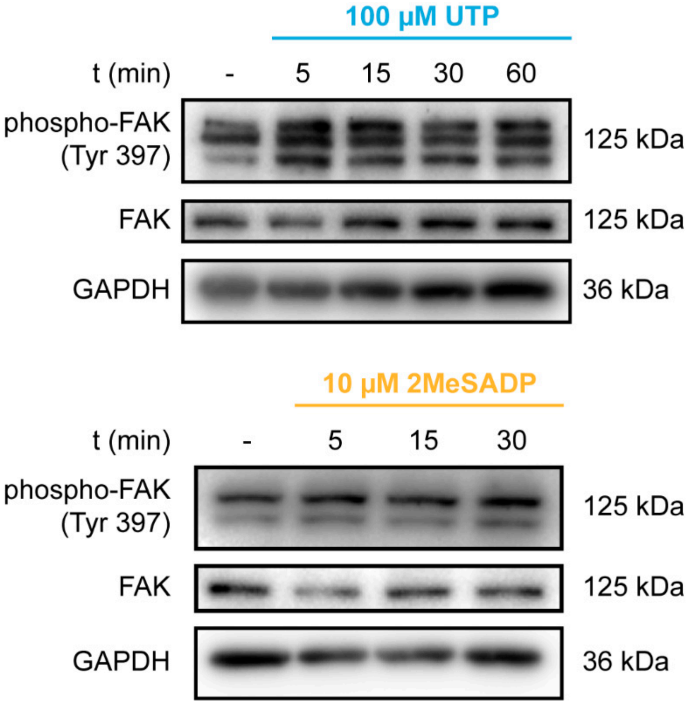

Figure S1. FAK phosphorylation induced by the UTP and 2MeSADP stimulation in rat cerebellar astrocytes. Representative blots showing changes in phopsho-FAK (Tyr 397) protein levels after treatment with either 100 µM UTP or 10 µM 2MeSADP for different periods of time are shown, together with total FAK and GAPDH blots.

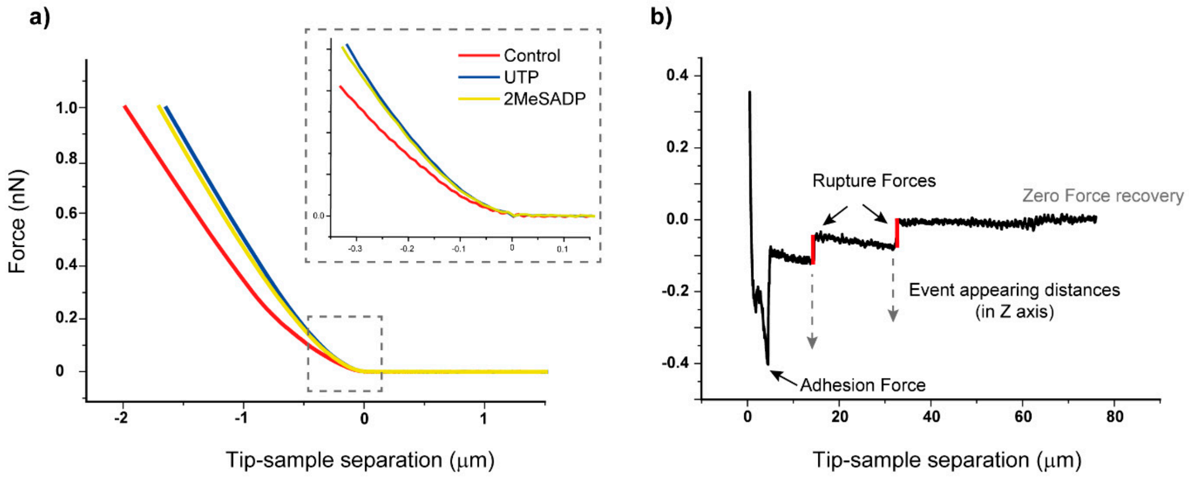

Figure S2. Representative examples of approach **(a)** and retract **(b)** plots obtained from Force measurements. Figure **(a)** shows the comparison between the average curves for astrocytes without (control, red) and with exposure to agonist molecules UTP (Blue) and 2MeSADP (yellow). The inset shows a magnified view of the area highlighted by the dashed grey box, which corresponds to the section of the curve employed for the calculation of the respective Young's moduli (initial 300 nm of the indentation). **(b)** Retraction plot showing the pulling distance-dependent adhesion-related factors (Adhesion Force, event rupture force and appearing distance). The rupture steps are highlighted in red.
